# Radiographic analysis of the thickness of the cranial bones in captive compared to wild-living cheetahs and in cheetahs with hypovitaminosis A

**DOI:** 10.1371/journal.pone.0255924

**Published:** 2021-08-10

**Authors:** Martin J. Schmidt, Gerhard Steenkamp, Peter Caldwell, Klaus Failing, Robert M. Kirberger

**Affiliations:** 1 Faculty of Veterinary Science, Department of Companion Animal Clinical Studies, University of Pretoria, Pretoria, South Africa; 2 Department of Veterinary Clinical Sciences, Small Animal Clinic, Justus-Liebig-University, Giessen, Germany; 3 Old Chapel Veterinary Clinic, Pretoria, South Africa; 4 Faculty of Veterinary Medicine, Unit for Biomathematics and Data Processing, Justus Liebig-University-Giessen, Giessen, Germany; University of Naples Federico II, ITALY

## Abstract

Captive cheetahs often demonstrate a high incidence of diseases in which vitamin A imbalances are implicated. These can occur even under controlled and optimised feeding regimens, which is why surveillance of vitamin A status is mandatory in the successful health management of cheetahs. Serum levels of the vitamin do not reflect the true vitamin A status and liver tissue analysis is rather impractical for routine application in large felids. A biomarker for evaluating overt and subclinical vitamin A deficiency in cheetahs is needed. This study evaluates whether increased calvarial bone thickness can be detected on routine skull radiographs of vitamin A deficient cheetahs compared to unaffected animals, and secondly, evaluates whether there is increased bone thickness in clinically sound captive cheetahs in general compared to wild-living controls. Bone thickness in the neuro- and splanchnocranium was measured in 138 skull radiographs. Significant thickening of the parietal bones was found in latero-lateral radiographs of immature cheetahs (< 12 months) with vitamin A deficiency. This finding may allow a presumptive diagnosis of hypovitaminosis A in immature cheetahs. A general difference in skull thickness between free-living and captive cheetahs was not found.

## Introduction

The cheetah is one of Africa’s most endangered large felids, threatened by habitat loss, poaching and killing to protect livestock outside reservation areas [1]. The population is at risk of further decline, which is why zoological gardens and wildlife centres are committed to conservation efforts, aiming to increase the genetic diversity of cheetah populations [2]. However, cheetahs can be a challenging species to manage in captivity, as they often demonstrate a high incidence of diseases in which, amongst others, vitamin imbalances have been implicated [3–10]. A stable vitamin A supply is important for captive cheetahs and other large felids because of their inability to convert precursors of the vitamin into the active form (retinol) [11,12]. Vitamin A plays an important role in cell division, cell differentiation and maintenance of differentiated cells in a number of tissues [13]. Hypovitaminosis A can lead to defective male and female fertility [8,14,15], disturbed skeletal growth [16] and osteoarthritis [17]. It has also been associated with central nervous system (CNS) malformations [3], stillbirth, weak new-born cubs and neonatal death [18,19], as well as degenerative myelopathy in cheetahs and other large felids [20,21]. Decreased vitamin A levels also negatively influence the differentiation of osteoclastic cells [22,23]. This has an impact on balanced bone growth and leads to hyperostosis of the skull bones, especially in lions, which can result in compression and damage to the adjacent brain and spinal cord [24], especially in immature animals, in which osteogenesis is dominant to achieve skeletal growth [25]. After attaining maturity, the effects of vitamin A deficiency are far less pronounced but can still have an impact on bone thickness and density [26].

Although zoos and rescue centres take great efforts to address nutritional requirements of cheetahs, vitamin A deficiency can occur even under controlled and optimised feeding strategies [27]. Besides clinically overt retinol deficiency that causes distinct clinical entities, a continuum of gradual vitamin A depletion has been described (subclinical or marginal deficiency), in which there is a diffuse impairment of various biochemical reactions in various organ systems before signs of classical deficiency may occur [28].

Due to the severe consequences of retinol deficiency, surveillance of vitamin A status is mandatory in their successful health management. Although vitamin A status can be assessed based on circulating retinol, it is known from humans and other mammals that serum retinol levels reflect the true general vitamin A status only when the systemic level is severely depleted or extremely high [13,29–31]. As the liver is the main storage site for vitamin A, measurement of hepatic concentrations is considered the most reliable assessment of vitamin A status in humans and animals [19,28]. This can be evaluated by liver biopsy, which carries the risk of potential procedural complications [32]. Given its invasive nature, liver tissue analysis is rather impractical for routine application in cheetahs, and would also not be feasible for population evaluation. Therefore, a reliable biomarker for evaluating clinically overt or subclinical vitamin A deficiency would be helpful.

In a study on dry skulls, an increased bone thickness was observed in captive compared to wild lions [33]. The authors suggested that a relationship between an increase in cranial bone thickness and insufficient vitamin A levels of the animals existed. This increase in bone thickness in captive vs free-living lions is also supported by observations of wildlife specialists with a focus on large felid diseases (personal communication P. Caldwell).

Based on the effects of vitamin A deficiency on skull thickness in lions, we hypothesise that skull bone thickness is also increased in captive compared to wild cheetahs in general, and even more pronounced in animals with clinically overt hypovitaminosis A. The aim of this study was to evaluate whether increased bone thickness can be detected in skull radiographs of vitamin A deficient cheetahs in comparison to unaffected animals, and secondly, to evaluate whether there is increased bone thickness in clinically sound captive cheetahs compared to wild-living controls.

## Materials and methods

### Animals studied

The picture archiving and communication system (PACS) of the Onderstepoort Veterinary Academic Hospital, Faculty of Veterinary Science, University of Pretoria and a private veterinary wildlife clinic (Old Chapel Veterinary Clinic) were retrospectively searched for digital radiographs of cheetahs from zoos and wildlife centres. Age, body weight and gender of cheetahs were recorded.

Skulls from wild cheetahs were obtained at general health checks in association with relocation programmes, as well as from museum collections in South Africa and Germany (Ditsong National Museum of Natural History, Pretoria, South Africa).

This study carried ethics approval from the University of Pretoria (REC107-18).

### Imaging techniques

#### X-ray equipment

Radiographs were made with different computed radiography systems (Kodak Point of Care CR360, Carestream Vita SE system and Fuji Capsula system). Exposure settings varied and depended on the age of the cheetah and body part examined. From museum specimens, latero-lateral head views were obtained using a CR system: (Apelum Baccara 90/20, Italy), using a Fuji CR console system (Fujifilm Medical Systems, Stamford, USA). All data were anonymised. Owner consent for use of clinical data was automatically obtained when owners agreed to the examination of the cheetahs.

#### Computed tomography (CT)

**Fig 1:**
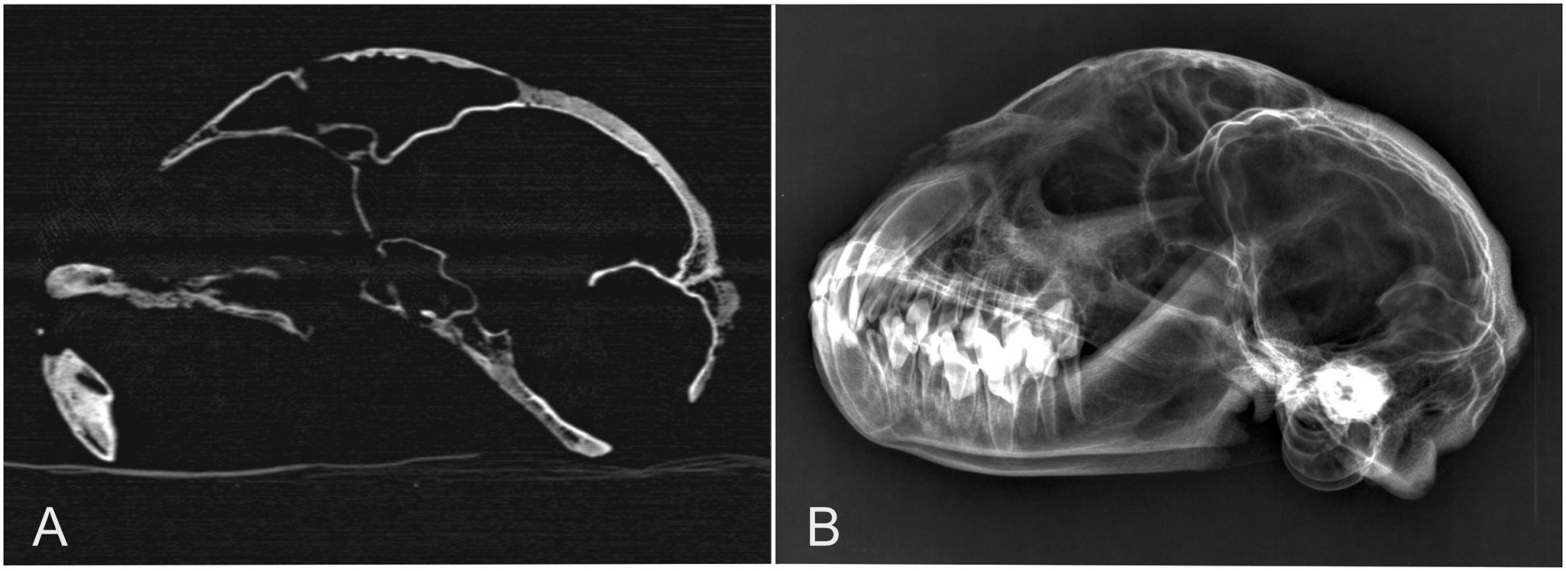
Midsagittal Computed Tomography in a bone window (A) and a corresponding latero-lateral radiograph of a cheetah skull (B).

Computed tomography data sets of 15 cheetah heads and dry skulls were acquired using a dual slice helical CT scanner (Siemens Somatom Emotion Duo, Siemens Medical Systems, Midrand, South Africa). These specimens were prospectively examined and were not part of the other analysis in this investigation, because of missing information concerning age, gender and body weight of the cheetahs. Technical settings included 120 kV, 350 mAs, matrix 512 x 512, slice thickness 0.8 mm and pitch 1 mm. Measurements of bone thickness obtained from radiographs of these specimens were compared to measurements obtained from CT images of the same skulls in order to assess the accuracy of radiographic measurements (Fig 1).

### Image analysis

Radiographs were given study numbers to blind interpreters to the cheetah’s origin and vitamin A status. Images with insufficient image quality and signs of other systemic diseases were excluded. Bone thickness in the neuro- and splanchnocranium was measured. In order to allow accurate repetitive measurements, special landmarks were defined, which were adopted from a previous MRI [25] and pathological study [34] and adapted to radiographic analysis.

### Latero-lateral view

**Fig 2:**
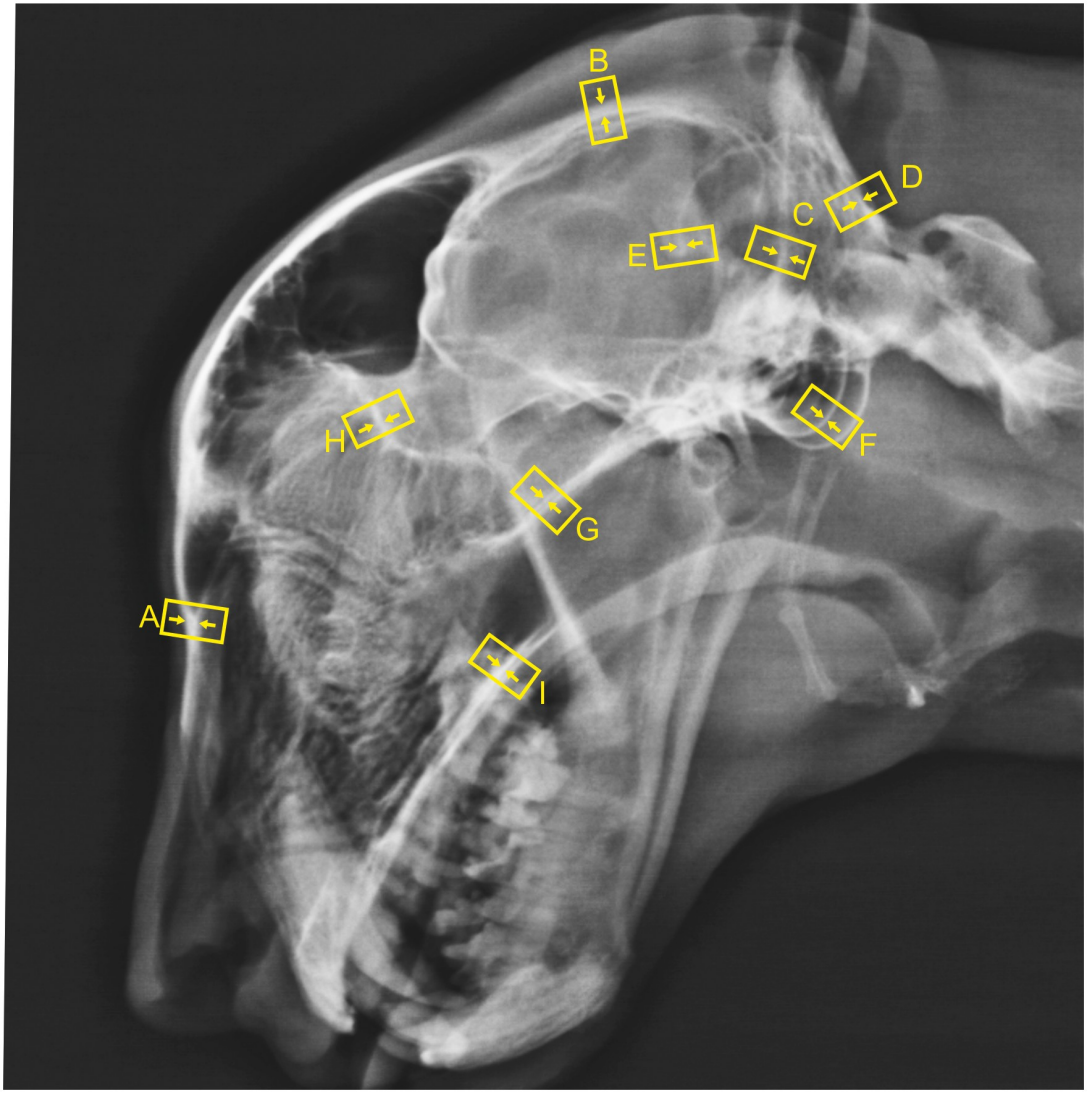
Latero-lateral radiograph of a four-year-old clinically sound captive cheetah displaying the measuring points of bone thickness: A: Nasal bone; B: Parietal bone; C: Nuchal crest; D occipital bone; E: Osseous tentorium; F: Tympanic bulla; G: Presphenoid bone; H: Cribriform plate; I: Palatine bone.

1. Thickness of the nasal bone at the middle kink (Fig 2A)
2. Thickness of the dorsal parietal bone bone/interparietal suture at its narrowest point (Fig 2B)
3. Maximum thickness of the nuchal crest (Fig 2C)
4. Minimum thickness of the occipital bone (Fig 2D)
5. Maximum thickness of the osseous tentorium (Fig 2E)
6. Maximum thickness of the tympanic bulla wall (Fig 2F)
7. Maximum thickness of the presphenoid bone (Fig 2G)
8. Maximum thickness of the cribriform plate (Fig 2H)
9. Thickness of the palatine bone measured directly behind the last molar tooth (Fig 2I)

In order to establish interobserver reliability, each parameter was measured by a diplomate neurologist (MJS) and diplomate radiologist (RK).

### Measurement of vitamin A

Hypovitaminosis A was diagnosed based on serum vitamin A levels. After centrifugation, plasma samples were stored at −20 °C until overnight shipment to a commercial laboratory. Samples were analysed within 48 hours after taking the blood. Analysis was performed by means of high-performance liquid chromatography (HLPC). Vitamin A levels included free retinol, protein bound retinol (holo-RBP), retinoic acid and retinyl stearate, -palmitate, and- oleate.

## Statistical analysis

### Bone thickness in captive vs fee-living cheetahs

All statistical analyses were performed using the statistical software package BMDP (BMDP Statistical Software, Inc., Los Angeles, USA). To evaluate the relationship between the measured bone thicknesses and body weight, age and gender, and to compare the adjusted means of bone thickness between captive and wild animals, a number of one-way analyses of covariance were performed. As the statistical distribution of the data for ‘thickness of presphenoid bone’ and ‘thickness of nasal bone’ was skewed to the right, and the relationship to the dependent variables was not linear, these values were transformed by logarithm. Within an analysis of covariance, the global dependency of the variables on the body weight, age and gender was tested. The slopes of the regression lines were compared, testing the null hypothesis that for a special dependent variable the slopes are identical. If slopes were statistically different (p < 0.05), differences in the increase or decrease of growth exist between groups. The increase (or decrease) of the dependant variable (slope) is presented for an increase of 1 kg body weight (BW) or one month increase in age.

In a second step, the adjusted means (sample means adjusted for a common mean body weight and a common regression line) of captive and wild cheetahs were calculated and checked for significant differences between groups. If significant statistical differences were given, the groups were compared pairwise using a t-test. Results of this calculation provide a comparison of the variables between groups corresponding to the same bodyweight given as a geometric mean at 33 kg.

### Bone thickness in cheetahs with diagnosed hypovitaminosis A

As the above-mentioned analysis revealed an influence of age on bone thickness, age matched groups were built for comparison between clinically normal (captive) cheetahs and captive animals with hypovitaminosis A. Normal distribution of these data was tested using the Shapiro–Wilk test. Differences in bone thickness values between the groups were tested for each parameter using t-tests. Statistical significance was again determined at an alpha level < 0.05.

### Accuracy of radiographic and CT measurements and intermodality variability

In order to assess a possible variability of the variables measured using radiography in contrast to measurement with CT, the accuracy of the measurements was determined using a Bland–Altman analysis, which considers the differences between the two methods for each parameter. The differences between the two measurements were plotted against the average of the two measurements. Accuracy was considered good if 95% of the differences were within two standard deviations (SD). P-values less than 0.05 were considered statistically significant. Interobserver variability was determined using the same method.

## Results

### Captive healthy cheetahs

One hundred and sixty-two skull radiographs from 142 cheetahs between May 2010 and February 2018 were reviewed. The majority of cheetahs came from the Ann van Dyk Cheetah Centre and the remainder came from private game farms in South Africa. All animals were accommodated in fenced bushveld camps in small groups. The animals were clinically healthy with normal reproductive status, and with no radiographic abnormalities. Radiographs were obtained under general anaesthesia as part of routine annual health examinations, or of traumatised cheetahs. Twelve of these cheetahs were examined twice, and four animal three times, all at different ages. Seventy-one images were rejected either because of poor radiographic quality or poor positioning of the skull. The radiographs of the remaining 91 cheetahs were allocated to the group of captive clinically sound cheetahs. Forty-two males and 41 females were evaluated whilst for eight, the gender was unknown. Their ages ranged from three to 182 months (mean 73.7 ± 44.14 months). Mean body weight was 33.1 kg ± 7.7 kg.

### Cheetahs diagnosed with hypovitaminosis A

Twelve captive cheetahs were identified with a diagnosis of hypovitaminosis A. Eight of the affected cheetahs were female, four were male. The mean age was 8.3 months (7–13 months). Mean body weight was 21±3.1. All cheetahs were in appropriate body condition. Clinical workup included physical and neurological examination, complete blood count, biochemistry panel and electrolytes. Clinical signs of hypovitaminosis included depressed mental status, ataxia of all four limbs, impaired balance, and opisthotonus. Three cheetahs had visual impairment that manifested as bumping into objects.

The mean serum vitamin A levels of the affected cheetahs were 0.2 ± 0.09 μmol/L (range 0.11–0.3) whereas in prior research reports clinically healthy cheetahs range is between 0.25–4.2 (Ø 1.82) μmol/L [11]. Clinical signs improved after vitamin A supplementation in affected animals.

Radiographs of ten immature cheetahs were used as a control group. None of these cheetahs had any characteristic signs of hypovitaminosis A. Age and body weight were not significantly different between the control vs study group (control group: 9 months, 19±5.1 kg vs. study group: 8.3 months; 21±3.9 kg). Vitamin A levels of these animals were not known.

### Free-living cheetahs

Twenty radiographs of free-living cheetahs were examined. Radiographs from living animals were obtained under general anaesthesia as part of routine annual health examinations, or of traumatised cheetahs. Twelve were males and eight were females. Their age was estimated based on skeletal characteristics [35] and ranged from six to 126 months (mean 61.4 ± 43 months). Data on bodyweight was available for 10 animals (mean 38.1 kg ± 9.3 kg).

#### Influence of age, gender and body weight on bone thickness

Evaluation of the captive clinically sound cheetahs revealed a high global dependency of age (p < 0.001) on all measured parameters. None of the measured parameters were influenced by gender or body weight. No significant differences for the equality of slopes of the measured parameters and body weight and gender were found. The slope of the regression line for the variables ‘thickness of the nasal bone’ and ‘thickness of palatal bone’ and body weight was significantly different for the wild cheetahs (p < 0.01 each), indicating a greater increase of thickness of the bones with body weight in the latter group (Table 1).

**Table 1:** Results of the one-way analysis of covariance and comparison of bone thickness in captive and wild cheetahs.

variable	covariate	group	Equality of slopes		Zero slopes		Equality of adjusted means	
			estimate	p-value	estimates	p-value	estimate (mm)	p-value
**Thickness nasal bone**	Age	captive cheetahs wild cheetahs	0.0039 0.01	**<0.01**	---	---	captive cheetahs: 1.002 (±0.092) wild cheetahs: 0.806 (±0.026)	**0.012**
	Gender	captive cheetahs wild cheetahs	0.07 0.12	0.74	0.07	0.18		
	BW	captive cheetahs wild cheetahs	0.005 0.004	0.072	(0.005)	(0.2)		
**Thickness of palatal bone (log)** ^§^	Age	captive cheetahs wild cheetahs	0.0001 0.0038	**<0.01**	---	---	captive cheetahs: 0.625 x (1.04^±1^) wild cheetahs: 0.48 x (10^±0.017^)	**0.0055**
	Gender	captive cheetahs wild cheetahs	−0.0044 0.064	0.62	0.006	0.85		
	BW	captive cheetahs wild cheetahs	0.0024 −0.002	0.27	0.0014	0.59		
**Thickness of the tentorium**	Age	captive cheetahs wild cheetahs	0.01 0.019	0.20	0.01	**<0.001**	captive cheetahs: 3.455 (±0.273) wild cheetahs: 2.651 (±0.106)	**0.009**
	Gender	captive cheetahs wild cheetahs	−0.078 0.297	0.66	−0.04	0.82		
	BW	captive cheetahs wild cheetahs	0.0225 0.0007	0.812	0.019	0.190		
**Thickness of occipital bone**	Age	captive cheetahs wild cheetahs	0.0145 0.0215	0.37	0.01	**<0.001**	captive cheetahs: 2.13 (±0.08) wild cheetahs: 2.04 (±0.22)	0.69
	Gender	captive cheetahs wild cheetahs	−0.1442 0.1985	0.74	−0.12	0.43		
	BW	captive cheetahs wild cheetahs	0.0056 −0.0284	0.69	0.0005	0.96		
**Thickness of the parietal bone**	Age	captive cheetahs wild cheetahs	0.0103 0.0152	0.18	0.01	**<0.001**	captive cheetahs: 3.048 (±0.09) wild cheetahs: 2.63 (±0.25)	0.13
	Gender	captive cheetahs wild cheetahs	0.1680 0.3319	0.15	0.19	0.29		
	BW	captive cheetahs wild cheetahs	0.0004 0.0284	0.55	0.005	0.68		
**Thickness of nuchal crest**	Age	captive cheetahs wild cheetahs	0.0002 −0.0028	**0.05**	---	---	captive cheetahs: 0.395 (±0.0114) wild cheetahs: 0.472 (±0.078)	0.35
	Gender	captive cheetahs wild cheetahs	0.0115 0.1011	0.08	(0.028)	(0.361)		
	BW	captive cheetahs wild cheetahs	−0.0005 0.0139	0.10	0.0025	0.28		
**Thickness of tympanic bulla**	Age	captive cheetahs wild cheetahs	−0.0001 −0.0002	0.92	−0.00008	0.78	captive cheetahs: 0.47 (±0.012) wild cheetahs: 0.438 (±0.034)	0.36
	Gender	captive cheetahs wild cheetahs	0.0041 0.0621	0.44	0.01005	0.67		
	BW	captive cheetahs wild cheetahs	0.0009 0.0009	0.86	0.00103	0.55		
**Thickness of presphenoid bone (log)** ^§^	Age	captive cheetahs wild cheetahs	−0.0007 −0.0001	0.82	−0.00062	0.31	captive cheetahs: 0.64 x (1.06^±1^) wild cheetahs: 0.64 (1.17^±1^)	0.98
	Gender	captive cheetahs wild cheetahs	−0.0039 0.117	0.52	0.0087	0.86		
	BW	captive cheetahs wild cheetahs	−0.0023 −0.0068	0.911	−0.0029	0.427		
**Thickness of the cribriform plate**	Age	captive cheetahs wild cheetahs	0.0012 0.0071	0.11	0.002	0.23	captive cheetahs: 1.22 (±0.08) wild cheetahs: 1.26 (±0.18)	0.86
	Gender	captive cheetahs wild cheetahs	−0.0075 0.278	0.43	0.029	0.84		
	BW	captive cheetahs wild cheetahs	−0.0046 −0.0017	0.29	−0.003	0.73		

The table presents results of the comparison of the equality of regression coefficients (slopes) on the body weight and the equality of adjusted means at BW = 33 kg. Significantly different estimates and p-values are in bold and underlined which represent the result of pairwise comparison. Normal typed estimates and p-values are not significantly different and represent the result of global comparison.

#### Influence of captivity on bone thickness

Results of the comparison of the adjusted means of the thickness of the osseous tentorium (p = 0.009), thickness of the palatal bone (p = 0.005) and thickness of the nasal bone (p = 0.012) were significantly different for captive and wild cheetahs. Comparison of the adjusted means of all other measured parameters revealed no statistically significant differences (Table 1).

#### Comparison of bone thickness between cheetahs with hypovitaminosis and age matched controls

**Fig 3:**
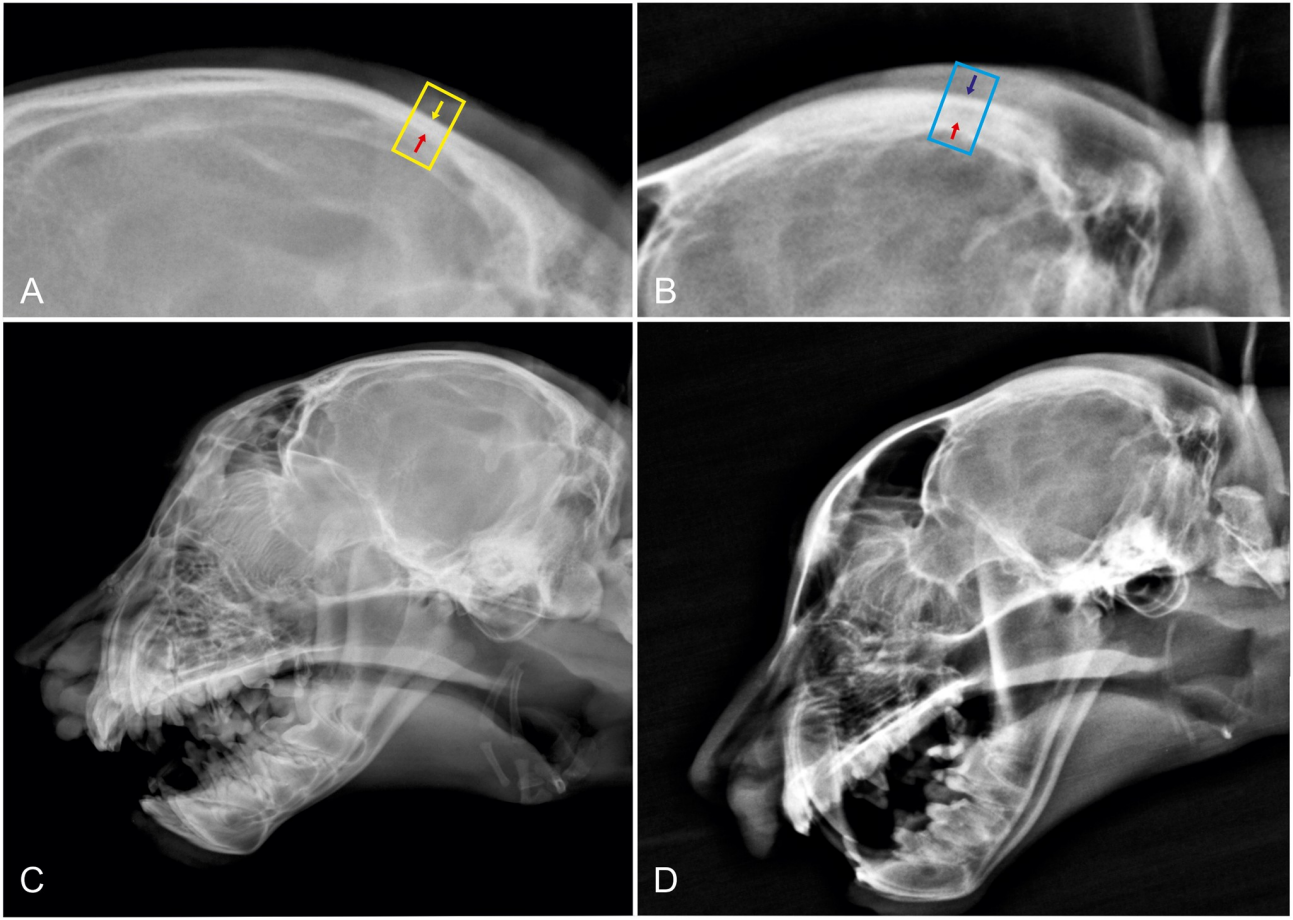
Parietal bone thickness in a healthy cheetah (A; C) compared to a cheetah with hypovitaminosis A (B, D). Red arrows mark the inner surface of the calvarial bones (*tabula interna*), the yellow and blue ones the outer surface (*tabula externa*) of the parietal bones in midline.

Results of the bone measurements of both groups are listed in Table 2. Bone thickness was significantly greater in the parietal bones (Fig 3) of cheetahs with hypovitaminosis A (p = 0.0002) but in no other measured value.

**Table 2:** Results of pairwise parametric (nasal bone, palatum thickness, occipital) and non-parametric testing of bone thickness at different measure points on the skull of clinically sound cheetahs and animals with overt hypovitaminosis A.

Group	Body weight	Age (months)	Thickness of nasal bone	Thickness of palatal bone	Thickness of osseous tentorium	Thickness of occipital bone	Thickness of parietal bone	Thickness of nuchal crest	Thickness of tympanic bulla	Thickness of presphenoid bone	Thickness of cribriform plate
**Hypo A (n = 12)**	21±3.1	8.3	0.76±0.045	0.62 (0.3–1)	3.3 (2.6–4)	1.32 (0.74–1.9)	**3.55 (2.8–4.6)**	0.6±0.1	0.6±0.07	1.2±0.29	1.2 (1–1.6)
**Control (n = 10)**	19±5.9	9	0.72±0.13	0.46 (0.3–0.6)	2.9 (2–4.3)	0.99 (0.6–1.3)	**2.67 (2.1–3.1)**	0.45±0.13	0.5±0.05	0.85±0.67	1.0 (0.5–2)
**p-value**	0.082	0.851	0.8	0.28	0.17	0.14	**0.0002**	0.19	0.128	0.557	0.85

Parameters with significant differences between groups are highlighted in bold type.

#### Accuracy of radiography vs CT

Ninety-five per cent of the differences between the CT and radiographic measurements were less than ± 2 SD from the mean difference for all variables, demonstrating good agreement between measurements except for the thickness of the presphenoid and palatal bones. The mean difference in these calculations was negative, indicating a tendency to measure higher values in radiography.

#### Interobserver accuracy

Ninety-five per cent of the differences between the first and second observer were less than ± 2 SD from the mean difference for all variables, demonstrating good agreement between measurements except for the measurement of the nuchal crest, the palatal and presphenoid bones. The mean difference was negative, indicating a tendency for the first (less experienced) observer (MJS) to measure higher values.

## Discussion

Vitamin A deficiency has been identified as a possible contributor to a number of health issues in captive cheetahs [6,17,27,36]. Despite nutrition with supplementary foods, captive felids are potentially subject to subclinical hypovitaminosis A [27]. The bioavailability of supplemented vitamin A and its absorption, metabolism and excretion may differ from a natural diet. Vitamin A deficiency can therefore occur even under controlled and optimised feeding strategies [17,27].

In bone, this vitamin is a potent stimulator of osteoclast formation and bone resorption [23], which is why vitamin A deficiency can lead to vertebral and calvarial hyperostosis and compression of nervous tissue. Excess vitamin A levels reduce cortical bone and skull thickness [37,38]. The changes in the skull and associated neurological deficits are well known problems in growing lions kept in captivity [3,18,25,32,39,41–43]. They were also found to a lesser extent in cheetahs [42]. Here, we evaluated whether cheetahs with clinically overt hypovitaminosis A showed increased thickness of their skull bones in radiographs. From all measured parameters of the skull, only the thickness of the parietal bone was significantly increased compared to that of age matched controls. This result is striking with respect to the fact that lions develop a massive hyperostosis of the entire neurocranium including frontal, parietal, supra-occipital, and basi-occipital bones, as well as the osseous tentorium, and cribriform plate [41]. Hypovitaminosis was diagnosed based on serum levels in the hypovitaminosis group. While normal serum values do not rule out hypovitaminosis, serum values below normal limits are usually diagnostic. Measurements based on liver samples would have been ideal, but were not available for the study. Although this might be a limitation, the association of classical clinical signs, low serum levels and improvement on clinical signs after vitamin A supplementation allow for a clear diagnosis of hypovitamonosis A in these cheetahs.

Extensive skull hyperostosis has also been experimentally produced in vitamin A deficient dog puppies [34]. The observed reduction in the activity of osteoclasts and consecutive hyperostosis varied dependent on the individual bone [34–36,39–43]. Larger bones with a large medullary cavity have higher metabolic rates, undergo a more intensive remodelling during growth [45,46]. These bones might be more prone to hyperostosis under deficiency conditions In most bones of the skull, the inner and outer tables of bone have fused to form a compact structure and the marrow cavity has been more or less eliminated. The parietal bones have medullary cavities, which might explain a selective hyperostosis in these bones, however, the medullary cavities of the occipital and basicranial bones are comparable or even larger, but did not show signs of enlargement in our study. Growth and remodelling processes can also be influenced by muscle strain [47]. Masticatory muscles broadly insert into the parietal bone, which may account for the observed selective increase in thickness in the parietal bones.

Failure to develop a more serious and widespread bony hypertrophy in hypovitaminosis A may reflect a generally slower rate of growth in the cheetah compared to lions [8–50]. A limitation of the measured result in the comparison between clinically healthy controls and vitamin deficient animals may be the limited number of examined cheetahs with hypovitaminosis. Examination of a larger group of cheetahs could also have revealed differences in other skull bones.

Studies that examined skulls in adult large felids demonstrated a significant difference in bone thickness between captive and free-living lions [33]. The most obvious lesions were thickening of the parietal bone, osseous tentorium of the cerebellum, dorsal arch of the atlas, basisphenoid bone and the occipital bone leading to a narrow foramen magnum, but all bones of the skull were affected [33,51–53]. Based on these findings, we also investigated whether an increased bone thickness occurs as a general phenomenon in captive cheetahs and if increased bone thickness revealed in skull radiographs might be used as a biological marker for the diagnosis of subclinical vitamin A deficiency. Although we found significantly increased bone thickness in the osseous tentorium, the palatal and nasal bones in captive cheetahs, the value of the results are disputable as differences ranged in the (sub-)millimetre level. This is again in contrast to findings in lions with hypovitaminosis A, in which skull bone thickness increased in centimetres [33,40]. Furthermore, interobserver variability was high for measurements of the palatal bone, putting the diagnostic value of this measurements into question. The reason for the minimal (or missing) differences between captive and wild cheetahs in comparison to the findings in lions is not clear. First, it must be considered that the vitamin A status was normal in the examined captive cheetahs. Vitamin A levels were not available for these animals, which is an important limitation of this study. However, none of these animals showed any clinical sign characteristic for vitamin A hypovitaminosis. Second, the possibility exists that there is a species-specific susceptibility of vitamin A deficiency in terms of variable vitamin A metabolism in bone tissue, or in general in large felids. The cheetah’s skeletal morphology shows a high grade of adaption for high speed [54,55] including a lightweight skeleton. The skull is smaller and thin boned compared to other large felids [56,57]. Control of bone growth in cheetahs may be subject to other regulatory principles than in lions, leading to very thin bones and less hyperostosis. Domestic kittens (3–6 months old) fed vitamin A deficient diets failed to develop osseous hypertrophy [58]. Other large felids also seem to be less or not susceptible to the effects of hypovitaminosis A on osseous tissue, at least findings of skull hyperostosis associated with hypovitaminosis A have never been reported [33,53,59–61]. Last, the majority of the cheetahs came from one research center, which might also have biased our results. Inclusion of radiographs from animals living in different centers all over South Africa would might have revealed differences between captive and wild animals, which is a further limitation of the study.

If the skull hyperostosis were less pronounced in cheetahs, other influential factors may have had an impact on the measurements and may have masked a possible difference. The influence of calorie intake on bone thickness can be ruled out, as we could not find an influence of body weight on skull thickness. None of the animals were neutered, which is why influence from an altered hormonal status can also be ruled out. The influence of gender on bone thickness was again not significant in our study. Inadequate intake of vitamin D may also influence growth and remodelling of bone tissue. To minimise this bias, cheetahs with radiological evidence of metabolic bone conditions were excluded from the investigation. However, subclinical differences cannot be completely ruled out.

A final factor to consider is the assumption that millimetre and submillimetre differences in skull bone thickness cannot be reliably measured using radiography. Superimposition of soft and hard tissue structures decreases the diagnostic value of conventional radiographs [62]. Structures closer to the X-ray source appear more magnified than those closer to the detector. Furthermore, projection geometry was not standardised for the radiographs, which all have a potential bias on the measurements. Deviations from the standard projection geometry and observer variability in landmark identification are considered potential sources of error [63]. However, images with insufficient quality were excluded and the good interobserver reliability for most measurements suggests that the parameters can be reliably employed in most regions of interest. Assessment of bone thickness should ideally be performed with cross sectional imaging techniques [40]. In our study, radiography led to a good accuracy in the determination when CT was used as the reference standard. Only the radiographic evaluation of the palatal and presphenoid bones led to an overestimation of the thickness and was significantly different from CT. Our findings imply that the radiographic measurements, taken under controlled conditions and ensuring true lateral positioning, could yield sufficiently accurate data compared to CT.

## Conclusion

Significant thickening of the parietal bones was found in latero-lateral radiographs of immature cheetahs with vitamin A deficiency. Using radiography as a biomarker may thus allow the early diagnosis of hypovitaminosis A in immature cheetahs.
